# Cellulases and beyond: the first 70 years of the enzyme producer *Trichoderma reesei*

**DOI:** 10.1186/s12934-016-0507-6

**Published:** 2016-06-10

**Authors:** Robert H. Bischof, Jonas Ramoni, Bernhard Seiboth

**Affiliations:** Austrian Centre of Industrial Biotechnology (ACIB) GmbH c/o Institute of Chemical Engineering, TU Wien, Gumpendorferstraße 1a, 1060 Vienna, Austria; Molecular Biotechnology, Research Area Biochemical Technology, Institute of Chemical Engineering, TU Wien, Gumpendorferstraße 1a, 1060 Vienna, Austria

**Keywords:** *Trichoderma reesei*, Cellulase, Recombinant protein production, Biorefinery, Lignocellulose, Gene expression, Consolidated bioprocessing

## Abstract

More than 70 years ago, the filamentous ascomycete *Trichoderma reesei* was isolated on the Solomon Islands due to its ability to degrade and thrive on cellulose containing fabrics. This trait that relies on its secreted cellulases is nowadays exploited by several industries. Most prominently in biorefineries which use *T. reesei* enzymes to saccharify lignocellulose from renewable plant biomass in order to produce biobased fuels and chemicals. In this review we summarize important milestones of the development of *T. reesei* as the leading production host for biorefinery enzymes, and discuss emerging trends in strain engineering. *Trichoderma reesei* has very recently also been proposed as a consolidated bioprocessing organism capable of direct conversion of biopolymeric substrates to desired products. We therefore cover this topic by reviewing novel approaches in metabolic engineering of *T. reesei*.

## Background

### A brief history of *T. reesei*

The most ancient biotechnological practices involving fungi for the production of beer, wine and cheese might date back several millennia, i.e. to the very beginning of literate civilization itself [1–3]. In contrast, the discovery of the filamentous mesophilic ascomycete *Trichoderma reesei* (then *Trichoderma viride*) for its astonishing potential to produce extracellular cellulases took place just over 70 years ago. Initially the destructive potential of the original *Trichoderma* sp. isolated from rotting US Army equipment on the Solomon Islands during World War II was seen rather problematic. Nevertheless, it was not long before researchers at the Natick Army Research Laboratories led by Mary Mandels and Elwyn T. Reese, the name giving researcher, sought to turn this problematic potential into purposive products [4]. In a screening of 14,000 moulds of the Quartermaster Collection *Trichoderma* sp. QM6a showed an outstanding ability to degrade native crystalline cellulose. The designation “QM6a” for the last remaining original *Trichoderma* isolate, which identified it as the sixth of six cultures of the fungus stored at the Quartermaster Collection at Natick, remained. This particular strain is not only regarded as the *T. reesei* reference strain, but also is the one strain from which all the mutants used in industry today have been derived.

Then and now, research on *T. reesei* was propelled by the idea that its secreted cellulases could have a game changing impact on the 200 year-old struggle to economically produce fuels from renewable, lignocellulosic biomass [5]. *T. reesei* research has since pioneered the concept of enzymatic saccharification of cellulose by a synergistic combination of different cellulase activities [6] and laid the groundwork for our current understanding of the regulation of the involved enzymes [7–9]. Its major cellobiohydrolase CBH1 (CEL7a) was also the first eukaryotic cellulase to be cloned and the first cellulase whose structure was solved [10, 11]. An important step towards applying *T. reesei* cellulases industrially was the development of efficient strain mutagenesis and screening procedures in the 1970s. In the following two decades, the titer of extracellular protein produced by the original strain QM6a could be increased by up to 20 fold through mutagenesis programs at Natick and Rutgers University. The latter culminated in the isolation of strain RUT-C30 [12, 13] (where “RUT” stands for Rutgers). Although the gold standard for cellulase production in industry was reported to be higher than 100 g/L, this strain still is the prototype cellulase hyperproducer available to the public domain [14] with titers of extracellular protein reaching 30 g/L on the cellulase inducing substrate lactose [15].

At first, however, other commercial applications for cellulases were developed [16–18] as it became clear that efficient and complete saccharification of lignocellulosic biomass to fermentable sugars requires many more enzymatic activities than initially anticipated. Again, research involving *T. reesei* continued to break new ground in the enzymology of both cellulose and hemicellulose degradation [19–25] and still does so today [26–28]. High-speed atomic force microscopy has now also visualized cellulose degradation demonstrating how the major cellobiohydrolase CBH1/CEL7A slides unidirectionally along the cellulose surface [29]. By the early 1990s, transformation techniques facilitating genetic engineering of *T. reesei* had become available [30, 31]. During the coming decade these technologies were instrumental in gaining new insights into the regulation of its enzymes and in altering the enzyme profile secreted by the fungus [32, 33]. At the time, *T. reesei* was also among the first hosts for the expression of mammalian proteins as exemplified by the expression of calf chymosin under *cbh1* (*cel7a*) expression signals [34].

By the end of the 1990s, Kuhls et al. [35] discovered that *Hypocrea jecorina* is in fact the sexual form of *T. reesei* which is the reason why a number of subsequent publications used *H. jecorina* as species name instead of *T. reesei*. A more detailed study on sexual development led to the hypothesis that strain QM6a is in fact female sterile [36] which could subsequently be linked to a mutation in the MAP-kinase scaffold encoding gene *ham5* [37].

**Fig. 1:**
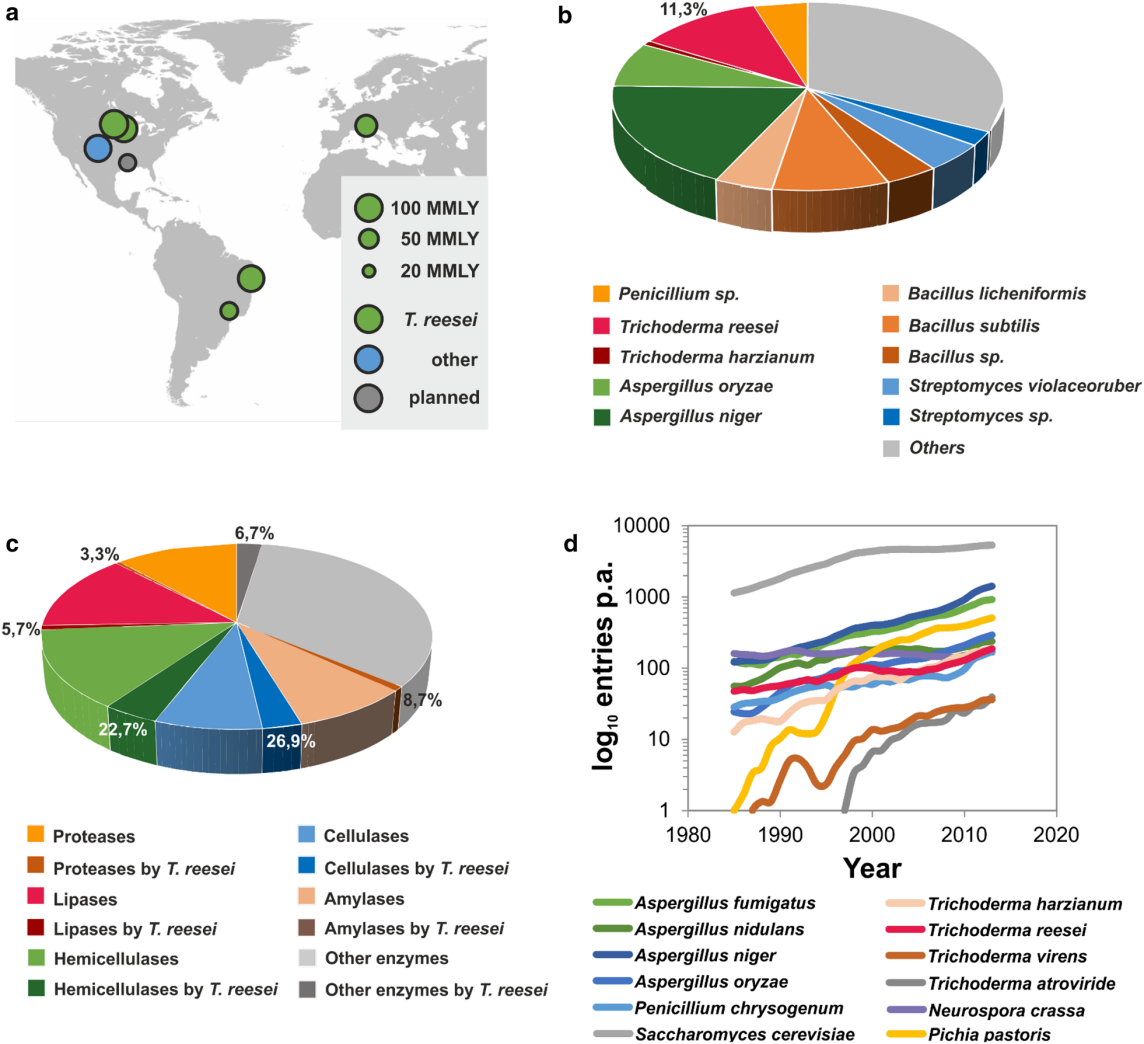
**a** Installed and planned cellulosic ethanol production as of April 2015 in million liter per year (MMLY). Capacity data were compiled from different specialist publications on cellulosic biofuels and press releases of involved consortia and companies. **b** Number of different technical enzyme preparations produced by individual species. **c** Number of a given type of enzyme produced by *T. reesei* (*darker color*) or other fungi (*lighter* *color*). In both cases (B + C) data were retrieved from the list of technical enzymes (2014 version) with kind permission from the Association of Manufacturers and Formulators of enzyme products (http://www.amfep.org). **d** Number of research papers per year for different fungi retrieved by a Scopus search with the species name as the entry. Results were averaged over 3 year intervals to reduce the effect of random fluctuation. When a second name for the species exists, control searches with both names were performed and the numbers compiled

The turn of the millennium, which for genetics can be viewed as a turn away from the study of isolated genes and pathways towards the study of entire genomes, saw the arrival of *T. reesei* in the so called Genomic Era. The first global approach to study *T. reesei* gene expression in 2003 was a transcriptomic study by Foreman et al. [38], who constructed DNA microarrays based on cDNAs that corresponded to over 5000 different transcripts of the *T. reesei* genome. Five years later, the genome sequencing and analysis of the original *T. reesei* isolate QM6a [39] laid the basis for the broad scale application of genome wide studies. In the following years, comparative genomic analysis of a number of cellulase hyper- and nullproducer strains led to the discovery of potential novel factors involved in cellulase hyperproduction such as nucleocytoplasmic transport, vacuolar protein trafficking and mRNA turnover [40–42]. These comparative analyses benefited from the fact that all *T. reesei* strains used in academia and industry are derived from strain QM6a. Sixty-five years after Elwyn Reese’s initial studies on cellulose degradation by *T. reesei* [43], the world-wide installed cellulosic biofuel production capacity is now 480.5 million liters per year (MMLY) of ethanol of which 380.5 MMLY (or roughly 80 %) are produced using *T. reesei* enzyme formulations such as Accellerase and Cellic (Fig. 1a). Without doubt, this effort required maturation of the underlying technology at several levels, including process engineering and substrate pretreatment. Still, the production and optimization of enzyme formulations for the biomass saccharification step was and remains to be one of the key factors determining the cost performance of cellulosic ethanol processes [44]. Moreover, enzyme production using *T. reesei* is by no means limited to the production of biorefinery enzymes. In fact, roughly 11 % of all technical enzyme formulations registered by the Association of Manufacturers and Formulators of Enzyme Products are produced using *T. reesei* as the expression host (Fig. 1b, c). Last but not least, *T. reesei* still maintains its importance in research, exemplified by more than 100 research articles dealing with the fungus or its enzymes published each year (Fig. 1d).

### The *T. reesei* biomass enzyme mix: new insights and limitations

In nature lignocellulose deconstruction is rarely accomplished by a single organism. It is rather achieved through the sequential order and collective effort of several organism that produce multiple carbohydrate-active enzymes (CAZymes) [45] to degrade the different polymers. Hence, it is not surprising that the secreted cellulase mix of *T. reesei* had to be significantly adapted to deliver cost competitive enzyme formulation for complete lignocellulose saccharification. Early on researchers realized that *T. reesei* formulations lacked sufficient β-glucosidase activity because most of the activity is bound to the fungal cell wall [46–48]. Consequently, increased β-glucosidase activity improved cellulose degradation because it counteracts product inhibition through cellobiose, which, in turn, is released by the cooperative action of endoglucanases and cellobiohydrolases [49]. This feedback mechanism otherwise seriously slows down cellulose saccharification. Likewise, hemicellulose-derived xylo- and mannooligosaccharides inhibit cellobiohydrolases of *T. reesei* [50–52] which strongly indicates that sufficient β-xylosidase and β-mannosidase activities are required to efficiently degrade lignocellulose. In 2003, Foreman et al. [38] described two proteins that are co-induced with the major cellulases and named them cellulose induced protein 1 and 2 (CIP1 and CIP2). They were since shown to be important to efficiently degrade lignocellulose [53]. Recent results show that CIP1 has structural similarities with lyases, although lyase activity could not be demonstrated [54], and that CIP2 is a glucuronoylesterase of the CE15 family [55]. Another important secreted protein is the swollenin SWO1 which contains a carbohydrate binding module (CBM) linked to an expansin like domain [56]. Despite cellulose disrupting activity [57, 58], SWO1 synergistically enhances endoxylanase rather than endoglucanase or cellobiohydrolase activity during enzymatic hydrolysis of pretreated corn stover [59]. One proposed mode-of-action is that it renders the xylan portion of lignocellulose more accessible for degradation by xylanases and thereby indirectly promotes the action of cellulases. Arguably the biggest revolution in recent years in cellulose degradation was the discovery of the lytic polysaccharide monooxygenases (LPMO). These enzymes introduced a new, oxidative mechanism to polysaccharide degradation. In cellulose degradation it is assumed that LPMOs act on the surface of crystalline cellulose fibrils, thereby rendering them more accessible to cellulases [60–62]. Intriguingly, these enzymes can derive the electrons needed for this process from plant cell wall lignin via long-range electron transfer, thereby turning the plant’s defense mechanisms against it [63]. Alternatively, GMC oxidoreductases or cellobiose dehydrogenases can work as electron donors [64]. This finding could well explain how *T. reesei* fuels its LPMOs as it was previously shown that several such GMC oxidoreductases are indeed induced by wheat straw [65]. However, this oxidative mechanism is by no means limited to cellulose depolymerization. Originally demonstrated for chitin [66, 67], LPMOs also play a role in xyloglucan [68] and amylose degradation [69]. In the CAZy database, enzymes belonging to this group have been reclassified to “auxiliary activities” (AA) as opposed to their previous classification as glycoside hydrolases (e.g. GH61) and are found in AA families 9–11 and 13 [45]. As outlined before, hemicellulolytic activities are important for complete lignocellulose saccharification (reviewed by Harris et al. [70]). Different amounts of individual activities are required depending on the hemicellulose types present in the substrate [71]. It is therefore noteworthy that the enzymatic repertoire of *T. reesei* has some clear limitations for certain types of hemicellulose specific linkages. One such missing activity is α-xylosidase. Adding α-xylosidase to a commercial *T. reesei* enzyme formulation enhanced the xylose and glucose release from pretreated corn stover [72]. Similarly, adding a GH family 5 cellulase with activity against glucomannan and xylan significantly improved a synthetic *T. reesei* enzyme preparation [73]. Other activities that are absent or very limiting in the *T. reesei* cellulase mix include endo-arabinase [74] and several pectinase activities [39, 75]. Supplementing commercial cellulase mixes with these enzyme activities consequently improved the saccharification of different substrates [76–78]. Another yet to be answered question is the in vivo function of secreted laccase-like multicopper oxidases encoded in the *T. reesei* genome [79].

### Improving *T. reesei* as protein production host

Given the fact that the cellulases and the majority of the other lignocellulose degrading enzymes are coordinately and conditionally expressed [33, 80], their transcriptional regulation represents a logical engineering target to improve cellulase production by the fungus. One of the master regulators is the carbon catabolite repression mediating C2H2 type transcription factor CRE1. CRE1 shuts down the transcription of its target genes when more favorable carbon sources such as glucose are present. Its truncation is one of the main causes for the improved cellulase production achieved by random mutagenesis programs of *T. reesei* QM6a leading to strain RUT-C30, which shows both a higher basal level and induced level of cellulase production [81–83]. Similarly, replacing CRE1 binding motifs in the promoter region of the major cellobiohydrolase *cel7a* with those of a known cellulase activator reduces carbon catabolite repression and elevates *cel7a* transcription under activating and repressing condition [84]. Additionally, the transcription of cellulase, xylanase and a number of other genes encoding enzymes involved in lignocellulose degradation strictly depends on the Zn(II)_2_Cys_6_ type transcriptional activator XYR1 [74, 85]. This is in contrast to other fungi including the Sordariomycetes *Neurospora crassa* and *Fusarium fujikuroi*, where the XYR1 orthologue exclusively modulates xylanase gene expression [80]. A mutation leading to a truncated form of XYR1 was found to cause the cellulase-negative phenotype of strain QM9136 which originates from the mutagenesis program at Natick [86]. Accordingly, cellulase over- and hyperproducing mutants have elevated levels of mRNA for the transcriptional activators *xyr1* [87]. It has also been proven that overexpression of XYR1 leads to a higher expression of cellulases and abolishes their catabolite repression in the presence of glucose [88, 89]. Furthermore, a point mutation within a putative regulatory region of XYR1 leads to a similarly deregulated expression pattern [90]. Besides XYR1 and CRE1, three transcription factors ACE1, ACE2 and ACE3 regulate cellulase and xylanase expression in *T. reesei* [91–93]. Similarly to CRE1, ACE1 is a C2H2 zinc finger repressor and its deletion therefore improves the production of both cellulases and xylanases [92]. ACE2 and ACE3, just like XYR1, are Zn(II)_2_Cys_6_ type transcription activators [87, 91, 93]. When *ace2* is absent, transcription of cellulases and xylanases is reduced accordingly, although cellulase induction by sophorose apparently remains unaffected [91]. Deletion of *ace3* completely abolishes cellulase transcription but merely reduces that of xylanases. While overexpression of ACE2 has not yet been attempted, overexpression of ACE3 leads to increased activities of both types of enzymes, as does the overexpression of six other so far uncharacterized regulators [93]. These include two further Zn(II)_2_Cys_6_ type transcription factors, as well as two WD40 proteins, a bromodomain protein and a *gcn5* related acetyltransferase. All three of the Zn(II)_2_Cys_6_ transcriptional activators (XYR1, ACE2 and ACE3) resemble the well characterized Gal4 protein of *S. cerevisiae.* It is well established that Gal4 recruits the Gcn5 containing SAGA complex [94, 95] and thereby promotes the transcription of its target genes through histone acetylation and euchromatin formation. As a matter of fact the Gcn5 orthologue of *T. reesei* is indispensable for cellulase expression and involved in the acetylation of histones in the *cbh1* promoter [96]. In recent years, a picture emerged which shows that the transcription of cellulases and related CAZymes in fungi is governed by a combination of many transcription factors representing a complex transcriptional-regulatory network influenced by counteracting activators and repressors. In *Penicillium oxalicum*, for instance, twenty transcription factors modulate the activation or repression of cellulase genes. Amongst these ClrB was identified as key integrator of all other regulators with their target genes [97]. Homologues of these regulators are found in *T. reesei*, but given the diversity of adaptations in plant cell wall regulation [80], it is expected that other and different regulators will also play significant roles. Another key player in cellulase regulation is the *T. reesei* orthologue of the enigmatic *Aspergillus* LaeA, which is involved in the regulation of secondary metabolite gene clusters in different fungi [98, 99]. While the deletion of this putative protein methyltransferase leads to a strong downregulation of different cellulase and other CAZyme genes, its overexpression can strongly promote their expression [88]. Similar effects were found for the LAE1 interacting VEL1 protein of the VELVET complex [100].

Most of the aforementioned studies provide fundamental insights into the regulation of cellulase formation. As most of these studies were carried out either in the original *T. reesei* isolate QM6a or the moderately overproducing strain QM9414, it remains unclear whether and to what extent these effects can be implemented in hyperproducing strains. In these strains processes such as translation, secretion and turnover of secreted enzymes, rather than transcription, might limit a further increase of cellulase production. It will be interesting to see whether or not several of the reported ways to improve cellulase gene expression can be stacked and how such strains would perform when compared to the hyperproducers derived by random mutagenesis.

Simply boosting the transcription of the gene of interest does not always lead to improved product formation especially in the case of non-fungal proteins. One successful strategy to circumvent low product formation is the fusion gene approach which uses besides promoter and terminator region of a highly expressed gene also the encoded protein as expression enhancer. For *T. reesei* this is the cellobiohydrolase encoding *cel7A* which is the most strongly expressed protein under cellulase inducing conditions [34]. It is believed that these gene fusions generally increase mRNA stability, import into the ER and passage through the secretory pathway. To this end the modular structure of CEL7A consisting of a catalytic module, a linker and a CBM is often exploited thereby replacing the C-terminal CBM by the gene of interest. Variations of this gene fusion approach are now available which target the protein to the ER for correct folding, disulfide bridge formation and glycosylation but subsequently aim for intracellular protein accumulation to avoid degradation of the desired product by extracellular proteases. One such strategy uses hydrophobins with an attached ER retention signal as carrier. These fusion proteins self-assemble to micelle-like structures and can be purified using a surfactant-based aqueous two-phase system [101]. A further strategy to target proteins to the ER uses the γ-zein peptide (ZERA) derived from the maize storage protein. Analogously, these self-assembling fusion proteins form protein bodies surrounded by ER membrane which protect them from proteolysis [102]. Developing fungi as efficient production hosts for mammalian proteins also requires the inactivation of the frequently encountered proteases in the fermentation broth. In a systematic study different secreted proteases related to degradation of biopharmaceuticals including antibodies, interferon α 2b, and insulin like growth factor were identified and several inactivated [103]. This led not only to a drastic reduction of protease activity but also to a strong increase of stability of all three recombinant proteins, with the antibody showing the most pronounced effect. Although engineering of the *N*-glycosylation pattern for the production of therapeutic proteins of high value was attempted before, creating an authentic human glycosylation pattern in a fungal expression host does not seem feasible at the moment. It is therefore questionable whether such biopharmaceuticals will be produced by fungal cell factories in the future, especially given the rapid development of CHO cells [104].

The above mentioned hydrophobins are another group of proteins which received considerable attention due to their surface-active properties [105]. These small, extracellular proteins self-assemble into protein layers at hydrophobic/hydrophilic interfaces due to their amphiphilic properties and make hydrophobic surfaces wettable or hydrophilic surfaces hydrophobic. They have a broad potential in food- and medical applications to disperse hydrophobic materials, stabilize foams or target different molecules to surfaces [106]. Cerato-platanins are another group of small, secreted proteins with four conserved cysteines. They bind to chitin and N-acetylglucosamine oligosaccharides and possess self-assembling properties at hydrophobic/hydrophilic interfaces. In contrast to hydrophobins, cerato-platanins rather enhance the polarity/apolarity properties of surfaces [107]. A targeting function is also attributed to CBMs present in different CAZymes. They can improve the hydrolytic activity of the catalytic domain they are attached to and lead to a more favorable pH and temperature optimum [108, 109]. Their carbohydrate binding properties can furthermore be exploited for affinity purification of fusion proteins using e.g. cellulose columns [110]. The broad range of other applications of recombinant CBMs has recently been reviewed elsewhere [111].

### *Trichoderma reesei* for consolidated bioprocessing and whole cell catalysis

**Fig. 2:**
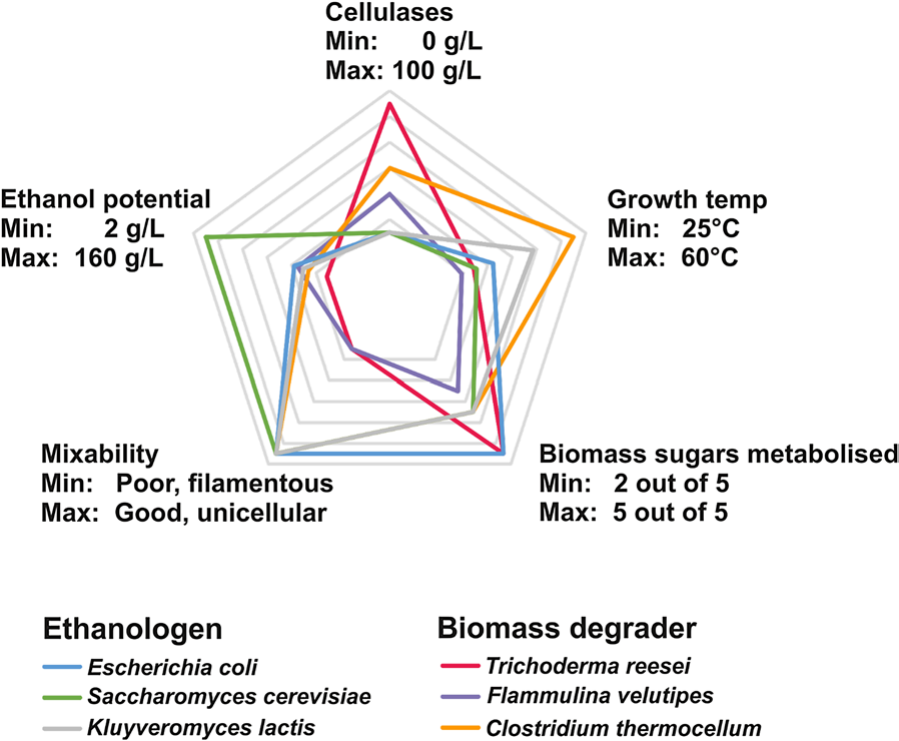
Radar chart showing the potential of different fungal and bacterial organisms as CBP organisms. Data were compiled from different reviews and original publications [123, 151–159]. The five biomass sugars are the hexoses glucose, mannose and galactose as well as the pentoses xylose and arabinose

Consolidated bioprocessing (CBP) is classically understood as the integration of the cellulolytic enzyme production, enzymatic hydrolysis and fermentation steps of a cellulosic ethanol production process into a single unit operation. Therefore, a single organism with good cellulolytic properties and an efficient ethanol fermentation pathway would be desirable. Nevertheless, using microbial consortia has also received some attention [112–114]. Unfortunately, no single organism that fulfils both requirements is currently available (Fig. 2). Consequently, efforts to engineer ethanologens to become cellulolytic [115, 116] or cellulolytic organisms to become ethanologenic [117, 118] have been undertaken. In the context of the first scenario, *T. reesei* has often served as a CAZyme gene donor, especially for *cel5a* and *cel7b* encoding two of its endoglucanases and for its two cellobiohydrolases *cel6a* and *cel7a* (Table 1). In all published studies the relatively low secretory capacity of *S. cerevisiae* limited substrate conversion by the secreted or membrane anchored heterologous CAZymes. Hence, high ethanol yields with realistic cellulosic substrates required supplementation with commercial *T. reesei* enzyme cocktails. Nevertheless, while efforts for improving the secretion and surface display capacity of engineered *S. cerevisiae* strains are now ongoing [119–121], the currently available cellulase displaying strains already have the potential to lead to substantial reductions of required enzyme loadings for biomass saccharification [122]. In the context of the second scenario, *T. reesei* itself represents a promising target organism. But although this fungus naturally possesses the ability to metabolize all the biomass related sugars and convert them to ethanol, yields are low and acetic acid is formed as an unwanted byproduct [123]. On the other hand, large scale fermentation regimes for *T. reesei* are well established due to its wide application in commercial enzyme production, and the molecular tools for its genetic engineering are very well developed too [124]. One of the remaining big challenges to employ *T. reesei* as a CBP organism is that several of its cellulases and glycolytic genes are repressed by hypoxia [125, 126], which is necessary for ethanol production. Additionally, the transcriptional repression of cellulases in the presence of ethanol [127] represents a further challenge to be overcome. However, the cellulase hyperproducing strain RUT-C30 has a higher ethanol tolerance as compared to strain QM9414 from the Natick lineage [127], and therefore represents a perfect platform strain to develop *T. reesei* as a CBP organism, especially since it is also carbon catabolite derepressed [81]. Furthermore, in the presence of a lignocellulosic pulp, RUT-C30 exhibits a pellet like morphology during the early stages of fermentation [128], which can be achieved in a growth independent manner by addition of the surfactant Triton X-100 and then also leads to a higher enzyme production [129]. This is important because poor mixability and low maximum cell densities as a consequence of filamentous growth so far hampered the use of *T. reesei* as a CBP organism. More generally speaking, consolidated bioprocessing could also be used to describe other integrated processes in which the substrate is not necessarily lignocellulosic biomass but another bio-polymer such as chitin or starch and the product is not ethanol, but any other metabolite [130]. To this end, a number of recent studies aimed at overproducing different metabolites via engineering of *T. reesei* (Table 2). However, the yields that could be achieved were in most cases far from commercialization, hence requiring further optimization.

**Table 1:** *T. reesei* genes used to engineer the ethanologenic yeast *S. cerevisiae* to a cellulose or hemicellulose degrader

Genes expressed^a^	Enzyme localization	Substrate	Ethanol (g/l)	Y_EtOH_ (% theory)^b^	Reference
*cel5a* + *bglA*_*A. aculeatus*_	Cell surface display (CSD)	45 g/l β-glucan	16.5	93	[160]
*xyn2* + *xylA*_*A. oryzae*_	CSD	100 g/l birchwood xylan	7.1	59	[161]
*cel6a, cel5a* + *bglA*_*A. aculeatus*_	CSD	10 g/l phosphoric acid swollen cellulose (PASC)	2.9	89	[162]
*cel7a*	Secretion	PASC, bacterial microcrystalline cellulose	–	–	[163]
*cel6a, cel5a* + *bglA*_*A. aculeatus*_	CSD	20 g/l PASC	7.1	75	[164]
*cel6a, cel5a* + *bglA*_*A. aculeatus*_	CSD	100 g/l pretreated rice straw	7.5	33	[164]
*cel6a, cel7a* + 20 other cellobiohydrolases	Secretion	20 g/l avicel cellulose	3	33	[165]
*cel6a, cel5a* + *bglA*_*A. aculeatus*_ + *swo1*	CSD	20 g/l PASC	3.4	36	[166]
*cel6a, cel5a* + *bglA*_*A. aculeatus*_ + *cdt1* _*N. crassa*_	CSD	20 g/l PASC	4.3	45	[115]
*cel6a, cel5a* + *bglA*_*A. aculeatus*_ + commercial cellulase	CSD	100 g/l avicel cellulose	48.9	87	[167]
*cel6a, cel5a* + *bglA*_*A. aculeatus*_ + commercial cellulase	CSD	200 g/l rice straw	42.2	86	[167]
*xyn2, xylA*_*A. oryzae*_ *, bglA*_*A. aculeatus*_	CSD	Slurry from rice straw pretreatment containing ~20 g/l poly-, oligo- and monomeric sugars	4.0	39	[151]
*cel5a* + *cbh1*_*T. emersonii*_ *fused to CBM of cel7a* + *bglA*_*A. aculeatus*_	CSD and secretion	10 g/l PASC	2.6 and 2.9	87 and 82	[168]

^a^ Genes are derived from *T. reesei* or from the species given in subscript

^b^ Based on a stochiometry of 0.51 g ethanol per g consumed sugar. Residual sugars in non-hydrolyzed substrates were considered when the data were available

**Table 2:** Examples for the genetically engineering of *T. reesei* towards overproduction of a metabolite or interesting molecule

Substance produced	Substrate employed	*T. reesei* strain background	Genetic alteration	Maximum titer	Reference
Ethylene	Wheat straw, cellulose	*T. reesei* (*viride*) TL124	Ethylene forming enzyme of *Pseudomonas syringae* expressed under the *cel7a* promoter	1.06 µl h^−1^ g^−1^ dry weight	[169]
Ethylene	Wheat straw	QM9414	Ethylene forming enzyme of *P. syringae* expressed under the *cel7a, pgk1* and *A. nidulans gpdA* promoters	4.012 nl h^−1^ l^−1^	[170]
N-acetyl-neuraminic acid	Chitin	QM9414	Expression of codon optimized N-acetyl glucosamine-2-epimerase from *Anabaena* sp. and N-acetyl neuramininc acid synthase from *Campylobacter jejuni* under the *pki1 and xyn1* promoters	13 µg g^−1^ mycelium	[171]
Xylitol	Barley straw	QM9414	Deletion of xylitol dehydrogenase, double deletion of xylitol dehydrogenase and l-arabinitol-4-dehydrogenase	13.2 g l^−1^	[172]
Xylitol	Xylose and glucose	QM9414	Knockdown (antisense RNA) of xylulokinase; deletion of xylitol dehydrogenase, overexpression of d-xylose reductase	3.7 g l^−1^	[173]
Erythritol	Wheat straw	RUT-C30, QM6a	Erythrose reductase expressed under the *pki1* and *bxl1* promoters	5 mg l^−1^	[174]

### Tools for cell design and engineering

While two comprehensive reviews on the molecular tool box of *T. reesei* and other *Trichoderma* species have only been given recently [124, 131], we want to update these with the most recent advances in the field. Targeted strain engineering towards improved cellulase production or for metabolic engineering requires efficient methods to introduce directed genetic alterations into the organism. The generally low efficiency of gene targeting has for a long time been a major challenge to obtain a reasonable number of transformants by homologous integration of a deletion or expression cassette. This problem has been mainly solved by inactivating components of the non-homologous end joining (NHEJ) pathway of DNA repair such as *tku70* [132] or *tmus53* [133]. *Tku70* deleted strains do show improved gene targeting although the efficiency of homologous integration in these strains can still vary depending on the targeted locus and thus might drop to 30 % [134]. Based on this improvement, a number of novel approaches were developed to insert expression cassettes at a defined genomic region, thereby avoiding pleiotropic effects caused by their random integration. Using a *tku70* background Jorgensen et al. [135] developed an expression platform that employs the easy to screen *ade2* locus as the preferred site of integration. Upon integration of the expression cassette into this locus, *ade2* is destroyed and the resulting transformants develop a distinct red pigmentation. In another study, the *pyr4* and *asl1* loci were chosen to develop a strain with uridine and l-arginine auxotrophy that allows site directed integration at these sites [136]. Ouaedraogo et al. [137] followed a different strategy and expressed the *S. cerevisiae* I-*Sce*I meganuclease in *T. reesei.* I-*Sce*I generates artificial double-strand breaks at an I-*Sce*I recognition site which was introduced beforehand at a predefined locus and improved both transformation and homologous integration efficiencies. In a follow up study I-*Sce*I mediated double strand breaks were combined with a *tku70* deletion [138]. Here, the inability to repair double strand breaks via NHEJ favors integration of the cassette leading to homologous recombination efficiencies of up to 100 %. A revolution for genetic engineering or genome editing was introduced with the CRISPR (clustered regularly interspaced short palindromic repeats)/Cas9 system [139]. Underscoring both the need of such a technology and the rising importance as an enzyme producer, this system was firstly tested for filamentous fungi in *T. reesei* [140]. It introduces specific DNA double strand breaks to stimulate gene targeting and depends only on a Cas9 (CRISPR associated) nuclease which uses a single chimeric guide RNA for targeting. Accurate targeting of this RNA-guided Cas9 to a specific DNA sequence is achieved by the protospacer sequence of the guide RNA via simple base pairing. Using 200 bp up- and downstream flanking regions for the gene deletion construct, HR frequencies of higher than 90 % could be reached. Double deletions and triple deletions occurred at a frequency of 45 and 4 %, respectively, following a single round of transformation. While in this study in vitro transcribed guide RNAs were cotransformed with the deletion cassette into a Cas9 expressing *T. reesei*, Nødvig et al. [141] used two flanking ribozyme sequences to liberate guide RNAs from a larger transcript that also encodes the Cas9 enzyme in different Aspergilli. Another essential tool needed in both recombinant protein expression and strain engineering are promoters that allow gene expression in a controllable fashion. Although a number of inducible and repressible promoters are available with the different cellulase promoter regions, they usually have drawbacks as their expression is linked to the host metabolism and activators might be limiting due to promoter titration effects. Useful alternatives include a set of l-methionine repressible *T. reesei* genes with different basal expression strength [65]. It was shown that one of these promoters can drive repressible expression of different reporter genes on several carbon sources including wheat straw. In a similar study, the promoter of a copper permease gene of *T. reesei* was used to control expression of the major cellulase and hemicellulase regulator *xyr1* in the absence of copper [89]. However, given the fact that copper containing enzymes from AA family 9 are important components of the *T. reesei* cellulase mix [142], it is, however, doubtful whether this system can be applied in a cellulase production scenario.

Beside these exciting molecular tools, some older tricks from classical genetics are now available too. Strain development of *T. reesei* was for a long time hampered by the fact that the fungus was thought to be asexual preventing strain crossing. A milestone in this respect was the finding that QM6a has a *MAT1*-*2* mating type locus and can be readily crossed with certain *MAT1*-*1T. reesei* wild-type isolates [36]. But when the *MAT1*-*2* locus of QM6a was replaced by its *MAT1*-*1* counterpart, no stromata were formed upon confrontation with the original *MAT1*-*2* QM6a strain. Consequently, it was impossible to exploit mating for strain engineering in the different academic and industrial *T. reesei* strains derived from QM6a. Using a systems biological approach the lacking gene responsible for female sterility was identified as *ham5* [37]. In *N. crassa, ham*-*5* encodes for a protein which serves as a MAP kinase scaffold during cell fusion [143, 144]. Reintroduction of a functional *ham5* restores stromata formation in strain QM6a and enables the restoration of female fertility in other strains originating from QM6a backgrounds [37]. This finding is especially important because crossing with the aforementioned *H. jecorina* isolates can lead to segmentally aneuploid progenies [145]. With this tool in hand, the basis for identifying relevant mutations leading to e.g. cellulase hyperproduction is laid. This is important as traditional complementation approaches to identify gene(s) causing mutant phenotypes were largely unsuccessful in species such as *T. reesei*. And although high-throughput sequencing and comparative genome analysis can easily identify mutations in the QM6a strain line of cellulase hyper- and nullproducers, only in a few cases this has already led to the linking of a mutation to a particular phenotype [86, 90, 146]. But in cases where a high number of mutations was found or the mutations affected genes with unknown function, comparative sequence analysis did not reveal the nature of the desired target genes [42]. Several researchers [147–150] have therefore successfully applied bulk segregant analysis in combination with next-generation sequencing to identify relevant mutations. In this approach, the mutant is crossed with a reference strain and the genomic DNAs of the segregants displaying the wanted phenotype are pooled and sequenced. Genome comparison of this pool of sequenced DNA to the genomes of the parental strains can then reveal conserved mutations relevant for the phenotype. Mutations which are unrelated to the phenotype will be underrepresented. Although it cannot be expected that this approach will lead to the identification of a single mutation because mutations close to the relevant mutation usually co-segregate, the number of targets for further investigation is considerably reduced.

## Conclusion

Research on *T. reesei* is highly driven by the industrial application of this fungus for the production of cellulases, other related CAZymes and recombinant proteins. Although a number of relevant key factors to improve protein production were discovered in the past decades, the underlying reason for its high potential to overproduce cellulases remained largely unidentified. Today, strain crossing in combination with different high throughput methods including comparative genomics in combination with tools such as the CRISPR/Cas9 system, provide efficient ways to pinpoint relevant mutations and thereby accelerate not only our understanding of cellulase regulation and expression but also to rational engineer and design this hyperproducing cell factory for a broader range of enzymes. Metabolic engineering of *T. reesei* for the direct conversion of lignocellulose to useful chemicals is still very much in its infancy. Future studies aimed in this direction will therefore need to push beyond a mere proof of principle and need to address and overcome inherent limitations of filamentous fungi as cell factories.

## Abbreviations

AA: auxiliary activities
Cas9: CRISPR associated protein 9
CAZymes: carbohydrate-active enzymes
CBM: carbohydrate binding module
CBP: consolidated bioprocessing
CHO: Chinese hamster ovary
CIP: cellulose induced protein
CRISPR: clustered regularly interspaced short palindromic repeats
CSD: cell surface display
GH: glycoside hydrolase
LPMO: lytic polysaccharide monooxygenase
MAT: mating type
MMLY: million liters per year
NHEJ: non-homologous end joining
PASC: phosphoric acid swollen cellulose
QM: quartermaster
MAP: mitogen-activated protein
